# Hospital Meetings

**Published:** 1896-05-16

**Authors:** 


### HOSPITAL MEETINGS.

### HAMPSTEAD HOSPITAL.

The biennial festival dinner of the Hampstead Hospital was held at the Grand Hotel, Northumberland Avenue, W.C., on the 7th inst. Mr. William F. Malcolm, the treasurer of the hospital, in the chair. Mr. Malcolm, in proposing the toast of the evening, made an excellent appeal on behalf of the hospital. He said that the change in the constitution admitting free patients which was made last year had necessarily added greatly to the expenses, so that though the hospital had received increased support its expenditure had outgrown its income. In round figures the hospital spent last year £2,750, of which £950 belonged to the nursing institution. Therefore the hospital itself spent £1,800 in 1895, while in the same year it received in ordinary income £1,200. This deficiency of £600 was reduced to about £400 through a legacy. The chief reasons why the hospital was a necessity were—first, the benefit received by the patients from the purer air of Hampstead; secondly, the risk of taking accidents to hospitals long distances away was avoided; and thirdly, patients' friends were able to visit them easily. Hampstead had a population of from 75,000 to 80,000 people, and it must be remembered that most provincial towns of the same size supported a local hospital. For these and other reasons he appealed to the wealthy residents of Hampstead to adequately support the hospital. The list of donations to the dinner fund included £150 from the chairman (whose list amounted to £1,235), £500 from Mr. Henry Harben, and £100 from Miss Harben. The total received was £1,617, an amount which Mr. Owthwaite, the able and energetic secretary, stated was the largest ever received.

### UNIVERSITY COLLEGE HOSPITAL.

H.R.H. the Duke of Cambridge presided at the festival dinner of the North London or University College Hospital, which was held at the King's Hall, Holborn Restaurant, on the 6th inst., and which was attended by a large number of influential friends of the hospital. His Royal Highness, in pleading the cause of the hospital, said that the institution required very considerable assistance, as although a great deal of good had been done by it, yet a good deal more could be done if funds were only forthcoming. A gathering such as that one could not, of course, individually get together a large sum, but all present had relatives and friends whom they could interest in the matter. He therefore wanted them, when they got home, or when they went on the following day to their ordinary avocations, to work amongst those friends and relatives on behalf of University College Hospital, with the result, he hoped, that the meeting would in the course of a few days, or weeks, or a month if necessary, go far towards getting rid of the very considerable debt—nearly £15,000—with which the hospital was burdened. Whilst the requirements of the hospital were increasing the income was decreasing, this latter fact being due in some measure, no doubt, to the prevailing craze for novelty. New hospitals nowadays were run after, whilst old and firmly-established ones like University were left to look after themselves. Mr. Henry Lucas (chairman of the Hospital Committee) contrasted the work done in 1885 with that done in 1895. The number of in-patients in 1885 was 2,673, and in 1895 2,980, or an increase of over 11 per cent.; while the out-patients numbered in 1885 35,170, and in 1895 44,201, or an increase of 26 per cent., the attendances of these cases amounting to 115,139 and 154,704 respectively, or an increase of 42 per cent. Despite this large increase in work done, the expenditure, owing to increased efficiency and economy, had decreased from £20,447 in 1885 to £19,337 in 1895. The Secretary (Mr. Newton H. Nixon) announced donations amounting to £1,424, and £22 in annual subscriptions.

### NATIONAL HOSPITAL FOR THE PARALYSED AND EPILEPTIC.

The annual meeting of the above hospital (held under the chairmanship of Mr. G. W. E. Russell on the 7th inst. at the hospital) was merely a formal one this year, at which the report and accounts were adopted and the usual votes of thanks passed. The result of the voting for eight pensions ranging from £5 to £20 was announced. The report for the year 1895 stated that 952 in-patients had been treated, and remained in the hospital on an average for 68 days each. 5,065 out-patients attended 32,119 times during the year. The widespread reputation of the hospital was illustrated by the number of applications for admission from distant places throughout the United Kingdom (patients even travelling to the hospital for treatment from foreign countries) in nearly every instance at the recommendation of local medical practitioners. Turning to the finances of 1895 we find that the ordinary receipts amounted to £9,108, whilst £14,074 was received from legacies, £12,860 was expended ordinarily, and £491 on extraordinary repairs. The large amount of legacies received has enabled the hospital to diminish the debt due to the bankers to the extent of £4,300 (leaving a balance due of £2,000) and to appropriate £5,400 to the purchase of the freeholds of Nos. 29 and 30, Queen Square. A point which we regret to notice is that while thirteen years ago annual subscriptions provided for 20 per cent. of the expenditure, at present this source of income only provides about 12 per cent.

### ROYAL BLIND PENSION SOCIETY.

The Royal Blind Pension Society, which was established in 1863 to give pecuniary aid to the blind poor at their own homes, held their festival dinner at the Whitehall Rooms on the 11th inst., under the presidency of H.R.H. the Duke of Cambridge. Throughout the evening his Royal Highness acted as his own toastmaster. Amongst those present were the Duke of Grafton (president of the society), the Earls of Chesterfield and Albemarle, Lords Amherst of Hackney and Colville of Culross, Sir Donald Currie, M.P., Sir W. T. Lewis, Bart., the Dean of Bangor, Admiral Sir Hunt Grubbe, and Lieut.-General Bateson. His Royal Highness pointed out that those deprived of sight were in a very pitiable condition. They must depend upon others to get them through life, and unless they had some means they were very helpless indeed. The question arose, what was the best way of rendering their life less burdensome than it naturally was, and what at the same time was the best way of making them useful members of the community? Undoubtedly the principle which underlay the work of the society, namely, helping them in their own homes. Turning to the growth of the society, his Royal Highness mentioned that twenty years ago the society paid only 200 pensions, amounting in the aggregate to £880. Ten years later there were 490, amounting to £3,277, and now the number of pensions exceeded 900, and involved the distribution of upwards of £6,000. The regular income derived from subscriptions was only £2,600. The society's plan of giving relief to the needy and deserving blind incurs no heavy administrative expenses. With the exception only of the secretary, its officers were all honorary. Subscriptions and donations to the amount of £2,532 were announced, including a special benefaction of £900.

